# Supplementary material for: The dual role of TonB genes in turnerbactin uptake and carbohydrate utilization in the shipworm symbiont Teredinibacter turnerae
Source: Appl Environ Microbiol. 2023 Nov 27;89(12):e00744-23. doi: 10.1128/aem.00744-23 (PMC10734418; doi:10.1128/aem.00744-23)
Supplement: Supplemental file 1 — Supplemental tables and figures, experimental details. [file aem.00744-23-s0001.docx]

**SUPPLEMENTARY MATERIALS**

The dual role of TonB genes in turnerbactin uptake and carbohydrate utilization in the shipworm symbiont *Teredinibacter turnerae*

Hiroaki Naka^a,b^ and Margo G. Haygood^a^#

^a^Department of Medicinal Chemistry, the University of Utah

^b^Division of Genetics, Oregon National Primate Research Center, Oregon Health & Science University

Running Head: The dual role of TonB genes in *Teredinibacter turnerae*

#Address correspondence to Margo G. Haygood, [mhaygood@ucsd.edu](mailto:mhaygood@ucsd.edu)

| **Table S1**. Strains and plasmids used in this study | | |
| --- | --- | --- |
| Strains and plasmids | Characteristics | Reference or source |
| ***T. turnerae strains*** |  |  |
| T7901 (ATCC 36867) | Wild-type strain isolated from *Bankia gouldi* | (1) |
| HNTT-1 | T7901∆*fttA* | This study |
| HNTT-2 | T7901∆*tnbF* | This study |
| HNTT-3 | T7901∆*tnbA*∆*tnbF* | This study |
| HNTT-4 | T7901∆*tnbA*∆*tnbF*∆*fttA* | This study |
| HNTT-5 | T7901∆*tonB1ab* | This study |
| HNTT-6 | T7901∆*tonB2* | This study |
| HNTT-7 | T7901∆*tonB3* | This study |
| HNTT-8 | T7901*∆tonB1ab∆tonB2* | This study |
| HNTT-9 | T7901*∆tonB1ab*∆*tonB3* | This study |
| HNTT-10 | T7901*∆tonB2*∆*tonB3* | This study |
| HNTT-11 | T7901*∆tonB1ab∆tonB2*∆*tonB3* | This study |
| HNTT-12 | T7901*∆tonB1ab∆tonB2*∆*tonB3* | This study |
| ***Escherichia coli* strains** |  |  |
| DH5α | ﻿F^−^, ϕ80*lacZ*ΔM15 *endA1* *recA1* *hsdR*17(r_K_−,m_K_+) *phoA* *supE*44 *thi*-1 *gyrA*96 *relA*1 Δ(*lacZYA-argF*)*U169* λ− | Laboratory stock |
| S17-1λ*pir* | λ-*pir* lysogen; *thi* *pro* *hsdR* *hsdM*^+^*recA* *RP4 2-Tc*::*Mu-Km*::*Tn7*(Tp^r^ Sm^r^) | (2) |
| π3813 | B462 Δ*thyA*::(*erm-pir-116*) (Erm^r^) | (3) |
|  |  |  |
| **Plasmids** |  |  |
| pGEM-T Easy | PCR products cloning vector, T-vector, Ap^r^ | Promega |
| pBluescript |  |  |
| pBBR1MCS-5 | broad host-range cloning vector, pBBR1, Gm^r^ | (4) |
| pDM4 | Suicide plasmid *sacB* gene, R6K origin, Cm^r^ | (5) |
| pEVS104 | Conjugation helper plasmid, R6K origin, *RP4,* *oriT,  tra, trb* and Km^r^ | (6) |
| pMMB208 | A broad-host-range expression vector; Cm^r^  *IncQ* *lacI*q Ptac; polylinker from M13mp19 | (7) |
| pPROBE'-gfp[ASV] | Promoter probe vector, pBBR1, gfp[ASV], Km^r^ | (8)  Addgene (40116) |
| pHN31 | pDM4 with the Km resistance gene | This study |
| pHN32 | pPROBE’-gfp[ASV] derivative, gfp[ASV] was replaced by lacIq-Ptac-polylinker from pMMB208 | This study |
| pHN33 | pHN32 derivative, the gentamicin resistance gene promoter from pBBR1MCS-5 was inserted. | This study |

| **Table S2**. Primers used in this study | |
| --- | --- |
| Primer name | Sequence (5’ to 3’) |
| **Mutagenesis** |  |
| **For ∆*fttA*** | |
| *fttA*-mut-*XhoI*-F | CTCGAGGAATGTGGGAAACACTCCACCTC |
| *fttA*-mut-*SmaI*-1 | GTACATGCTTACGCCGGGCTAGAACCCGGGAAACATCTAAACCCCCGGTAAATC |
| *fttA*-mut-*SmaI*-2 | GATTTACCGGGGGTTTAGATGTTTCCCGGGTTCTAGCCCGGCGTAAGCATGTAC |
| *fttA*-mut-*SpeI*-R | ACTAGTGCGATGGACCACATCACAAACTGTG |
| **For ∆*tnbA*** | |
| *tnbA*-mut-*SacI*-F | GAGCTCTAAAGTGGCGCTACAGTGCATTTCAAC |
| *tnbA*-mut-*SmaI*-1 | TTAAGTCTTCAGGCTGAGAGTGTAGCCCCGGGATTTAATGCTTCGACGAGGCTGTTCATG |
| *tnbA*-mut-*SmaI*-2 | ATGAACAGCCTCGTCGAAGCATTAAATCCCGGGGCTACACTCTCAGCCTGAAGACTTAAC |
| *tnbA*-mut-*XhoI*-R | CTCGAGAATCTGGGTGCGCTCTAAATCGATTTC |
| **For ∆*tnbF*** | |
| *tnbF*-mut-*XhoI*-F | CTCGAGCTCTTCGATCAGTCGCGCAGG |
| *tnbF*-mut-*SmaI*-1 | CTCACACGTTGGCGAGCTGCACCCCGGGGAGTGTGTGCGATGTCATCAGATG |
| *tnbF*-mut-*SmaI*-2 | CATCTGATGACATCGCACACACTCCCCGGGGTGCAGCTCGCCAACGTGTGAG |
| *tnbF*-mut-*SpeI*-R | ACTAGTGAACCGTGTTCGAACAACTGGATAG |
| **For ∆*tonB1ab*** | |
| *tonB1ab*-mut-*XhoI*-F | CTCGAGAATCGGTTTAGATTCTTCACTCTTGTGC |
| *tonB1ab*-mut-*SmaI*-1 | GCACTGCTGACTTGCAGAATTTACCCGGGCATCAGCGGGACTCCTTGTCCTTAAG |
| *tonB1ab*-mut-*SmaI*-2 | CTTAAGGACAAGGAGTCCCGCTGATGCCCGGGTAAATTCTGCAAGTCAGCAGTGC |
| *tonB1ab*-mut-*SpeI*-R | ACTAGTACATCTACTCCTGCATAGTTAATCAC |
| **For ∆*tonB2*** |  |
| *tonB2*-mut-*XhoI*-F | CTCGAGTGTTTGGTACAACGTTGATTAACCG |
| *tonB2*-mut-*SmaI*-1 | CATAAATATCACCCTGCCGCTACCCGGGCATCGCTCACCTACCTGTTTTC |
| *tonB2*-mut-*SmaI*-2 | GAAAACAGGTAGGTGAGCGATGCCCGGGTAGCGGCAGGGTGATATTTATG |
| *tonB2*-mut-*SpeI*-R | ACTAGTGCCATCTTCACTGCGATATTGATG |
| **For ∆*tonB3*** |  |
| *tonB3*-mut-*XhoI*-F | CTCGAGACCATTCCTACCATGGCGGGCATG |
| *tonB3*-mut-*EcoRV*-1 | CACGCATCGCTCTCCTCCTCGTTAGATATCCATCAATTACTGGCCTCCCTCGGCTGC |
| *tonB3*-mut-*EcoRV*-2 | GCAGCCGAGGGAGGCCAGTAATTGATGGATATCTAACGAGGAGGAGAGCGATGCGT |
| *tonB3*-mut-*SpeI*-R | ACTAGTGCTCCCAATAGGTCGCCTTGTC |
|  |  |
| **Complementation** |  |
| **For *fttA*** |  |
| *fttA*-com-*KpnI*-F | GGTACCGATTTAATAGTATAACTTTATCGCTTATCG |
| *fttA*-com-*EcoRI*-R | GAATTCCTAGAAGCTATAAGTCGCACTCAAATAC |
| **For *tonB1a*** |  |
| *tonB1a*-com-*KpnI*-F | GGTACCGAATATCGCCCGTTTGAAGGCGCTTAAG |
| *tonB1a*-com-*EcoRI*-R | GAATTCTCAGCCTTCGAGGTTAAATACAAACG |
| **For *tonB1b*** |  |
| *tonB1b*-com-*KpnI*-F | GGTACCTTAACCGGCACTGTTGTACGTCAATCAC |
| *tonB1b*-com-*EcoRI*-R | GAATTCTTAGTCCTTCATCACAAAGGTGAGGC |
| **For *tonB2*** |  |
| *tonB2*-com-*XbaI*-F | TCTAGAGTGTTGGGGATGTCTCTGTCTC |
| *tonB2*-com-*SacI*-R | GAGCTCCTACTTGTCTTTGGCCATTTGGAAAG |
| **For *tonB3*** |  |
| *tonB3*-com-*KpnI*-F | GGTACCGGCGCGGAAAGTGTGTCTGTCGCAG |
| *tonB3*-com-*EcoRI*-R | GAATTCTTAGCGGCTCAGTTTAAATTCGATCG |
|  |  |
| **qPCR** |  |
| *gyrB*-qPCR-F | AGGCTTACCGGGGAAATTGG |
| *gyrB*-qPCR-R | CCTTTAAGCGGCAAGATCGC |
| *fttA*-qPCR-F | AGCAACACTGACCTGGAACC |
| *fttA*-qPCR-R | CTGCTCGCGGTAATCTTTGC |
| *tnbA*-qPCR-F | GAACGCACCATTCAAGGCTC |
| *tnbA*-qPCR-R | ATCGGATGCGAGAAACAGCA |
| *tnbF*-qPCR-F | ATCTGGAGAGCCACAACAGC |
| *tnbF*-qPCR-R | ATTTGGGTGAGCAGGGTGAG |
| *tonB1a*-qPCR-F | GCGACCTATCCCGCACTTTA |
| *tonB1a*-qPCR-R | AACCCGCTGTACAGTAGCAC |
| *tonB1b*-qPCR-F | GGAGGAGCCCAAGCTTGTTAT |
| *tonB1b*-qPCR-R | AGATGGGTGTCATCCGCTTG |
| *tonB2*-qPCR-F | TGCGGCACTGGTAACTCTTG |
| *tonB2*-qPCR-R | AATTTCCCGCTCAGGCATCA |
| *tonB3*-qPCR-F | GCCGAAGTTCTGGAAGCTGA |
| *tonB3*-qPCR-R | TGAGCCTTCGGCATACCATC |

**RNA-sequencing**

*T. turnerae* T7901 was grown in non-aggregation SBM broth medium containing sucrose supplemented with 10 µM FAC (iron rich condition) or 0.1 µM FAC (iron limiting condition), and the expression of genes were compared by RNA-sequencing (RNA-seq). We avoided using an iron chelator to exclude any effects other than iron chelation caused by iron chelators.  2 biological replicates were used.

Illumina library construction using the Illumina TruSeq Stranded Total RNA Library Prep Kit with Ribo-Zero (Illumina) and sequencing using Novaseq 6000 with 150 bp paired-end runs was performed at the Huntsman Cancer Institute’s High-Throughput Genomics Center at the University of Utah. All raw sequencing reads were deposited in the NCBI Sequence Read Archive (SRA) under the accession number PRJNA885807.

Quality control and preprocessing of FASTQ reads were performed using the fastp program (9). The processed reads were mapped on the chromosome of *T. turnerae* T7901 using HISAT2 (10) and the output was converted to bam files using SAMtools (11).  FeatureCounts (12) were used to count the reads on *T. turnerae* T7901 genes, and differential expression analysis was carried out using the EdgeR package (13).

| **Table S3.** RNA-seq analysis to understand iron regulation of iron transport genes | | | | |
| --- | --- | --- | --- | --- |
| **Turnerbactin biosynthesis and transport cluster** | | | |  |
| Locus tag | gene name or annotation | logFC | logCPM | FDR-adjusted p-value |
| TERTU_RS18025 | *fttA* | 8.55 | 12.09 | 2.94E-64 |
| TERTU_RS18030 | hypothetical protein | 7.13 | 7.10 | 1.42E-66 |
| TERTU_RS18035 | hypothetical protein | 7.39 | 7.23 | 9.20E-66 |
| TERTU_RS18040 | PepSY domain- containing protein | 6.19 | 9.74 | 9.50E-46 |
| TERTU_RS18045 | *tnbC* | 11.52 | 12.90 | 5.89E-93 |
| TERTU_RS18050 | *tnbE* | 12.27 | 13.20 | 5.32E-98 |
| TERTU_RS18055 | *tnbB* | 12.72 | 12.92 | 4.30E-98 |
| TERTU_RS18060 | *tnbA* | 11.24 | 11.69 | 2.03E-88 |
| TERTU_RS18065 | efflux RND transporter periplasmic adaptor | 10.83 | 11.60 | 1.20E-72 |
| TERTU_RS18070 | multidrug efflux RND transporter permease | 6.9 | 12.44 | 1.84E-44 |
| TERTU_RS18075 | esterase | 8.41 | 10.40 | 5.04E-65 |
| TERTU_RS18080 | MbtH domain protein | 9.61 | 9.60 | 5.90E-76 |
| TERTU_RS18085 | *tnbF* | 8.41 | 13.36 | 3.25E-60 |
| TERTU_RS18090 | *tnbS* | 8.56 | 11.54 | 1.23E-62 |
| **TonB1 cluster** |  |  |  |  |
| Locus tag | gene name or annotation | logFC | logCPM | FDR-adjusted p-value |
| TERTU_RS04350 | transcriptional regulator | 0.31 | 2.96 | 0.44 |
| TERTU_RS04355 | *tonB1a* | 1.17 | 5.86 | 2.55E-03 |
| TERTU_RS04360 | *tonB1b* | 0.33 | 3.93 | 0.49 |
| TERTU_RS04365 | *exbB1* | -0.59 | 3.12 | 0.1 |
| **TonB2 cluster** |  |  |  |  |
| Locus tag | gene name or annotation | logFC | logCPM | FDR-adjusted p-value |
| TERTU_RS01630 | *ttpB2* | -1.03 | 11.08 | 0.01 |
| TERTU_RS01635 | *ttpC2* | -0.81 | 11.59 | 0.05 |
| TERTU_RS01640 | *exbB2* | -0.74 | 10.02 | 0.09 |
| TERTU_RS01645 | *exbD2* | -0.76 | 9.50 | 0.09 |
| TERTU_RS01650 | *tonB2* | -0.51 | 9.78 | 0.26 |
| TERTU_RS01655 | *ttpD2* | -0.5 | 10.93 | 0.28 |
| **TonB3 cluster** |  |  |  |  |
| Locus tag | gene name or annotation | logFC | logCPM | FDR-adjusted p-value |
| TERTU_RS08980 | TonB-dependent receptor | -0.42 | 4.39 | 0.21 |
| TERTU_RS08985 | *ttpB3* | -1.61 | 1.06 | 3.69E-04 |
| TERTU_RS08990 | *ttpC3* | -0.81 | 2.55 | 0.03 |
| TERTU_RS08995 | *exbB3* | -0.84 | 1.82 | 0.03 |
| TERTU_RS09000 | *exbD3* | -0.6 | 1.65 | 0.16 |
| TERTU_RS09005 | *tonB3* | -1.12 | 2.54 | 2.20E-03 |
| TERTU_RS09010 | *ttpD3* | -0.6 | 4.11 | 0.07 |

logFC, log fold change; LogCPM, log counts per million; FDR-adjusted p-value, False Discovery Rate-adjusted p-value

| **Table S4** RNA-seq result to understand iron-regulation of genes potentially encoding TonB-dependent outer membrane receptor | | | |
| --- | --- | --- | --- |
|  |  |  |  |
| Locus tag | logFC | logCPM | FDR-adjusted p-value |
| TERTU_RS00400 | -0.50 | 5.33 | 0.16 |
| TERTU_RS02235 | -2.90 | 13.53 | 3.18E-08 |
| TERTU_RS02920 | 0.02 | 9.41 | 0.97 |
| TERTU_RS03510 | -0.02 | 6.33 | 0.97 |
| TERTU_RS05530 | -1.72 | 5.88 | 1.14E-08 |
| TERTU_RS05545 | 2.88 | 10.49 | 7.93E-13 |
| TERTU_RS05620 | -0.22 | 4.95 | 0.56 |
| TERTU_RS06590 | -0.85 | 5.08 | 0.01 |
| TERTU_RS06645 | -3.05 | 7.41 | 2.40E-14 |
| TERTU_RS06655 | 0.03 | 12.84 | 0.95 |
| TERTU_RS06660 | -1.03 | 6.60 | 1.06E-03 |
| TERTU_RS08920 | 0.27 | 5.13 | 0.49 |
| TERTU_RS08980 | -0.42 | 4.39 | 0.21 |
| TERTU_RS09115 | 1.65 | 7.14 | 6.99E-04 |
| TERTU_RS10285 | -1.59 | 9.61 | 1.45E-03 |
| TERTU_RS10395 | -0.26 | 6.34 | 0.47 |
| TERTU_RS11325 | 6.33 | 12.22 | 2.48E-44 |
| TERTU_RS11935 | -1.37 | 11.73 | 5.43E-04 |
| TERTU_RS12735 | -1.83 | 4.66 | 2.04E-05 |
| TERTU_RS12820 | -0.13 | 6.73 | 0.76 |
| TERTU_RS14810 | 0.13 | 10.38 | 0.79 |
| TERTU_RS14845 | 0.60 | 6.81 | 0.10 |
| TERTU_RS14930 | -0.15 | 7.46 | 0.72 |
| TERTU_RS15340 | -5.98 | 9.09 | 7.14E-19 |
| TERTU_RS15625 | -2.16 | 5.58 | 5.97E-06 |
| TERTU_RS16015 | -1.26 | 8.11 | 8.80E-04 |
| TERTU_RS16355 | 0.44 | 7.52 | 0.24 |
| TERTU_RS16735 | 1.66 | 8.63 | 1.45E-05 |
| TERTU_RS17025 | -1.76 | 8.63 | 2.25E-05 |
| TERTU_RS17140 | 3.44 | 8.38 | 1.94E-19 |
| TERTU_RS17895 | 4.09 | 9.96 | 2.41E-20 |
| TERTU_RS18025 | 8.55 | 12.09 | 2.94E-64 |
| TERTU_RS18165 | -2.09 | 5.48 | 7.17E-13 |
| TERTU_RS18190 | -1.39 | 4.11 | 1.30E-05 |
| TERTU_RS18495 | -2.11 | 7.59 | 9.63E-07 |
| TERTU_RS19320 | 0.40 | 5.09 | 0.23 |
| TERTU_RS20475 | 0.76 | 4.31 | 0.02 |
| TERTU_RS20690 | -0.72 | 12.24 | 0.09 |

**Figure S1**. Structures of bacterial siderophores discussed


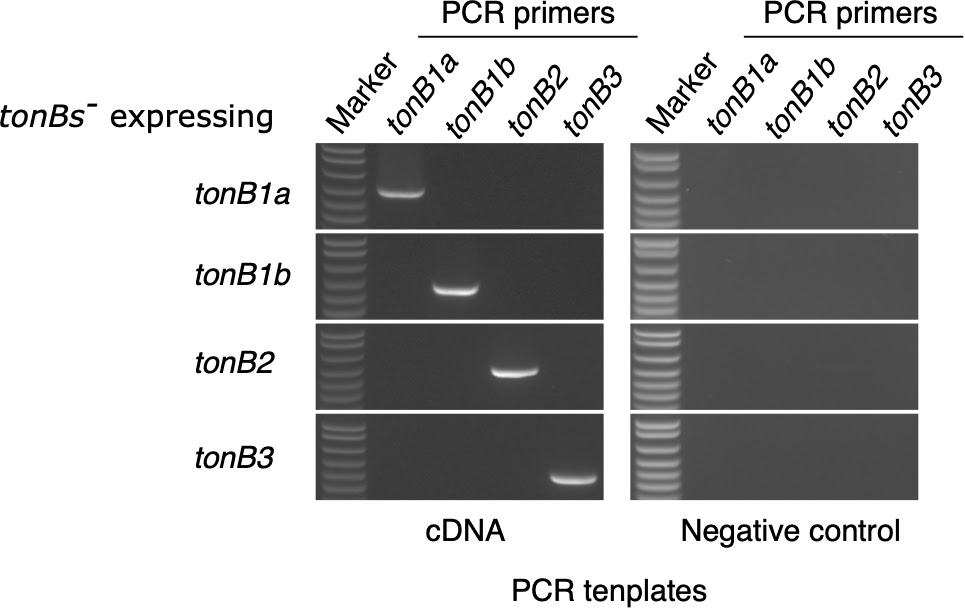


**Figure S2**. RT-PCR to confirm the expression of TonB genes

Each TonB gene was cloned into the expression vector pHN45 and conjugated into the *T. turnerae* T7901 ∆*tonB1ab*∆*tonB2*∆*tonB3* (tonBs-) strain in which all *tonB* genes were deleted. The strains were grown in SBM medium containing sucrose (0.5%), FAC (10 µM) and Km (50 µg/ml) at 30°C until reaching the exponential phase (OD_600_ 0.2-0.3). Negative control, RT-reaction without RT enzyme; FAC, ferric ammonium citrate; pHN45, plasmid expression vector.

**Fur titration assay**

The fur titration assay (FURTA) was performed as described by (14). DNA fragments to be tested were cloned into pBluescript II and transformed into *E. coli* H1717, and transformants were streaked on MacConkey agar plates supplemented with ammonium iron (II) sulfate (30µM) and Amp (100 µg/ml). Appearance of pink color (Lac+ phenotype, Fur-binding to cloned DNA fragments) around streaked *E. coli* was checked after overnight incubation at 37°C.


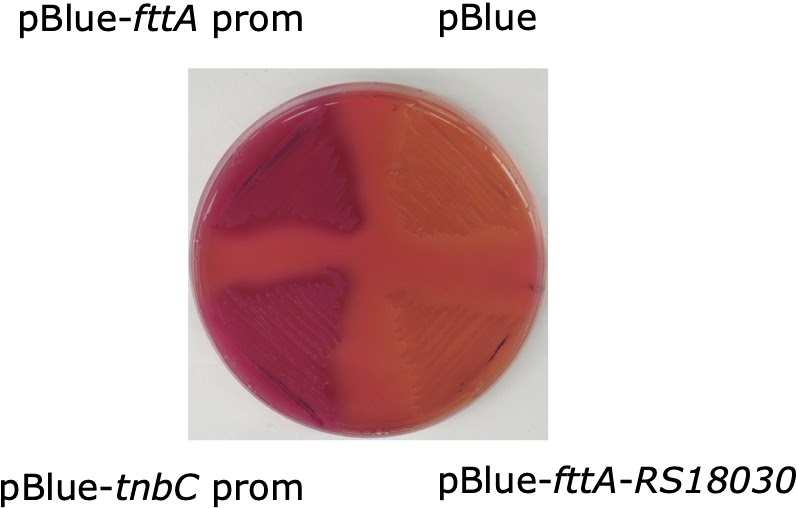


**Figure S3**. *E. coli* Fur can bind to the promoter regions of *fttA* and *tnbC*.

Binding of *E. coli* Fur to promoter regions of *fttA*, *tnbC*, and the region between *fttA* and *TERTU_RS18030* (negative control) was tested by Fur titration assay (14). *E. coli* H1717 containing plasmids harboring each fragment were streaked on MacConkey agar plates, and the presence of pink color (Lac+ phenotype) around streaked bacteria was evaluated after overnight incubation at 37°C. pBlue, pBluescript II; *fttA* prom, *fttA* promoter region; *tnbC* prom, *tnbC* promoter region; *fttA*-*RS18030*, the intergenic region between *fttA* and *TERTU_RS18030*.

**REFERENCES**

1. Distel DL, Morrill W, MacLaren-Toussaint N, Franks D, Waterbury J. 2002. Teredinibacter turnerae gen. nov., sp. nov., a dinitrogen-fixing, cellulolytic, endosymbiotic gamma-proteobacterium isolated from the gills of wood-boring molluscs (Bivalvia: Teredinidae). Int J Syst Evol Microbiol 52:2261-2269.

2. Simon R, Priefer U, Pühler A. 1983. A broad host range mobilization system for in vivo genetic engineering: Transposon mutagenesis in gram negative bacteria. Bio/Technology 1:784-91.

3. Le Roux F, Binesse J, Saulnier D, Mazel D. 2007. Construction of a Vibrio splendidus mutant lacking the metalloprotease gene vsm by use of a novel counterselectable suicide vector. Appl Environ Microbiol 73:777-84.

4. Kovach ME, Elzer PH, Hill DS, Robertson GT, Farris MA, Roop RM, 2nd, Peterson KM. 1995. Four new derivatives of the broad-host-range cloning vector pBBR1MCS, carrying different antibiotic-resistance cassettes. Gene 166:175-6.

5. Milton DL, O'Toole R, Horstedt P, Wolf-Watz H. 1996. Flagellin A is essential for the virulence of Vibrio anguillarum. J Bacteriol 178:1310-9.

6. Septer AN, Wang Y, Ruby EG, Stabb EV, Dunn AK. 2011. The haem-uptake gene cluster in Vibrio fischeri is regulated by Fur and contributes to symbiotic colonization. Environ Microbiol 13:2855-64.

7. Morales VM, Backman A, Bagdasarian M. 1991. A series of wide-host-range low-copy-number vectors that allow direct screening for recombinants. Gene 97:39-47.

8. Miller WG, Leveau JH, Lindow SE. 2000. Improved gfp and inaZ broad-host-range promoter-probe vectors. Mol Plant Microbe Interact 13:1243-50.

9. Chen S, Zhou Y, Chen Y, Gu J. 2018. fastp: an ultra-fast all-in-one FASTQ preprocessor. Bioinformatics 34:i884-i890.

10. Kim D, Paggi JM, Park C, Bennett C, Salzberg SL. 2019. Graph-based genome alignment and genotyping with HISAT2 and HISAT-genotype. Nat Biotechnol 37:907-915.

11. Li H, Handsaker B, Wysoker A, Fennell T, Ruan J, Homer N, Marth G, Abecasis G, Durbin R, Genome Project Data Processing S. 2009. The Sequence Alignment/Map format and SAMtools. Bioinformatics 25:2078-9.

12. Liao Y, Smyth GK, Shi W. 2014. featureCounts: an efficient general purpose program for assigning sequence reads to genomic features. Bioinformatics 30:923-30.

13. Robinson MD, McCarthy DJ, Smyth GK. 2010. edgeR: a Bioconductor package for differential expression analysis of digital gene expression data. Bioinformatics 26:139-40.

14. Stojiljkovic I, Baumler AJ, Hantke K. 1994. Fur regulon in gram-negative bacteria. Identification and characterization of new iron-regulated Escherichia coli genes by a fur titration assay. J Mol Biol 236:531-45.
